# Effect of implantoplasty on the elastic limit of dental implants of different diameters

**DOI:** 10.1186/s40729-021-00363-6

**Published:** 2021-08-24

**Authors:** Markel Diéguez-Pereira, David Chávarri-Prado, Iratxe Viteri-Agustín, Oier Montalban-Vadillo, Esteban Pérez-Pevida, Aritza Brizuela-Velasco

**Affiliations:** 1 Department of Surgery and Medical-Surgical Specialties, Faculty of Dentistry, University of Oviedo, C/ Catedrático Jose María Serrano s/n, 33006 Oviedo, Spain; 2 Department of Pharmacology and Physiology, School of Medicine, University of Zaragoza, 50009 Zaragoza, Spain; 3 Department of Surgery, Faculty of Medicine, University of Salamanca, Campus Miguel de Unamuno, 37007 Salamanca, Spain

## Abstract

**Background:**

Implantoplasty reduces both implant diameter and the thickness of its walls, subsequently reducing the ability of the implant to resist fracture in response to functional load. In combination with an increase in the crown-implant ratio due to bone loss, this could increase the lever effect, which in presence of high masticatory forces or parafunctional habits, could lead to complications such as fracture of the implant or loosening of the prosthetic screw.

**Objectives:**

To determine the elastic limits of internal connection, dental implants of different designs and diameters after an implantoplasty.

**Materials and methods:**

This in vitro study included 315 tapered internal connection titanium dental implants, the threads of which were removed with an industrial milling machine—for standardized implantoplasty (IMP1; *n* = 105)—or with the conventional approach—manually, using high-speed burs (IMP2; *n* = 105). The remaining 105 implants were used as controls. The final implant diameters were recorded. The quality of the newly polished surfaces was assessed by scanning electron microscopy. All implants were subjected to a mechanical pressure resistance test. A Tukey’s test for multiple comparisons was used to detect differences in the elastic limit and final implant diameters between the implant groups.

**Results:**

There were statistically significant differences in the elastic limit between the IMP1, IMP2, and control groups (*p* < 0.05). Furthermore, the implant diameter was significantly smaller in the IMP1 and IMP2 groups (*p* < 0.05). Scanning electron microscopy revealed smooth implant surfaces in the IMP1 and IMP2 groups, with some titanium particles visible in the IMP1 group.

**Conclusions:**

Implantoplasty significantly decreased the elastic limit of internal connection titanium dental implants, especially in those with a smaller diameter (3-3.5 mm).

## Background

The dental implants replacing missing teeth have been reported to have a long-term survival rate of 96.4% [1]. However, they may be associated with esthetic, mechanical, or biological complications, including mucositis and peri-implantitis [2]. Mucositis affects only the peri-implant mucosa and does not involve the marginal bone, whereas peri-implantitis is a pathological plaque-associated condition characterized by inflammation of the peri-implant mucosa and subsequent progressive loss of supporting bone [3]. Peri-implantitis is an important cause of implant failure, affecting up to 18.5% of treated patients and 12.8% of all dental implants placed, according to a recent meta-analysis [4].

Despite numerous reports on the treatment of peri-implantitis in recent years, there is lack of a universally accepted treatment [5–8]. The mechanical, non-surgical treatment seems to be ineffective [8–11], and by far, surgical treatment is considered the best option for the treatment of moderate or severe cases [12].

Resective procedures for peri-implantitis focus on facilitating the removal of granulation tissue and halting the progression of the disease, while maintaining a functional implant. Regenerative treatments have also been proposed for restoration of the original peri-implant bone [13].

The main objectives of treatment for peri-implantitis are the removal of calculus and biofilm attached to the implant and to prevent the adhesion of a new biofilm. Therefore, incomplete decontamination of the implant surface could be the biggest obstacle in maintaining the remaining peri-implant bone [14, 15]. A rough implant surface facilitates greater bacterial adhesion and colonization, which influences the formation and maturation of the biofilm [16–19]. Therefore, implantoplasty has been investigated as a technique for decontamination and prevention of bacterial re-colonization. Implantoplasty is a surgical procedure, where implant threads are removed and the surface is smoothed and polished using a rotary instrument [18, 20–24]. When performed as part of surgical treatment, implantoplasty achieves favorable clinical results and stabilizes bone loss [18, 20]. However, several problems have been identified while using this technique. For example, use of high-speed rotary instruments can increase peri-implant temperature and affect the surrounding tissues [7, 15], though this problem can be solved by adequate irrigation. Another problem could be the dispersion of titanium particles in adjacent tissues [8, 21, 25]. Implantoplasty reduces both the diameter of implant and thickness of its walls [11, 19, 24], thereby reducing the ability of the implant to resist fracture in response to functional load. In combination with an increase in crown-implant ratio due to bone loss, this could increase the lever effect, which in presence of high masticatory forces or parafunctional habits, could lead to complications such as fracture of the implant or loosening of the prosthetic screw [19, 24, 26, 27].

The objective of this in vitro study was to compare the elastic limit of dental implants across different designs and diameters when subjected to implantoplasty.

## Methods

The study included 315 self-tapered implants with either a “tissue level” internal connection design with 1.5 mm long machined collar, 3.5 mm or 4 mm wide body diameter, and prosthetic platform with a diameter of 4.5 mm (Klockner Essential Cone, Escaldes Engordany, Andorra) or a “bone level” internal connection design with 3 mm, 3.5 mm, or 4 mm wide body diameter and prosthetic platform with a diameter of 2.7 mm, 3.2 mm, or 3.4 mm, respectively (Klockner Vega, Escaldes Engordany, Andorra). The sample size was calculated using the Piface Version 1.76 software. After considering a significance level of 0.05, a margin of error of 0.05 and a confidence interval of 95%, 17.31 specimens were considered sufficient to detect differences between the groups in this study. After adjusting the sample for possible losses, the sample was established at 21 specimens per group.

The implants had a total length of 10 mm, and a moderately rough surface that had been achieved by sandblasting and acid etching of the surface. Sixty-three implants of each design and diameter were randomly divided into an industrially machined implant (IMP1) group (*n* = 21), a manually polished implant (IMP2) group (*n* = 21), and a control implant group (*n* = 21). Ethical approval was not required for this in vitro study. The study was conducted in compliance with the Standards for Reporting Qualitative Research guidelines, which were accessible through the EQUATOR network.

### Implantoplasty procedure

In both IMP1 and IMP2 groups, the apical 7 mm of each implant was fixed in a resin block with 4.5 mm (tissue-level implants) and 3 mm (bone-level implants) of implant left outside, where 1.5 mm of machined collar (for tissue-level implants only) and 3 mm (for both tissue-level and bone-level implants) of treated surface simulated marginal bone loss of 3 mm. The resin block had a modulus of elasticity ≥ 3 GPa, which was similar to that of the bone. A cover screw was placed on the implant to protect the implant connection from titanium debris.

**Fig. 1:**
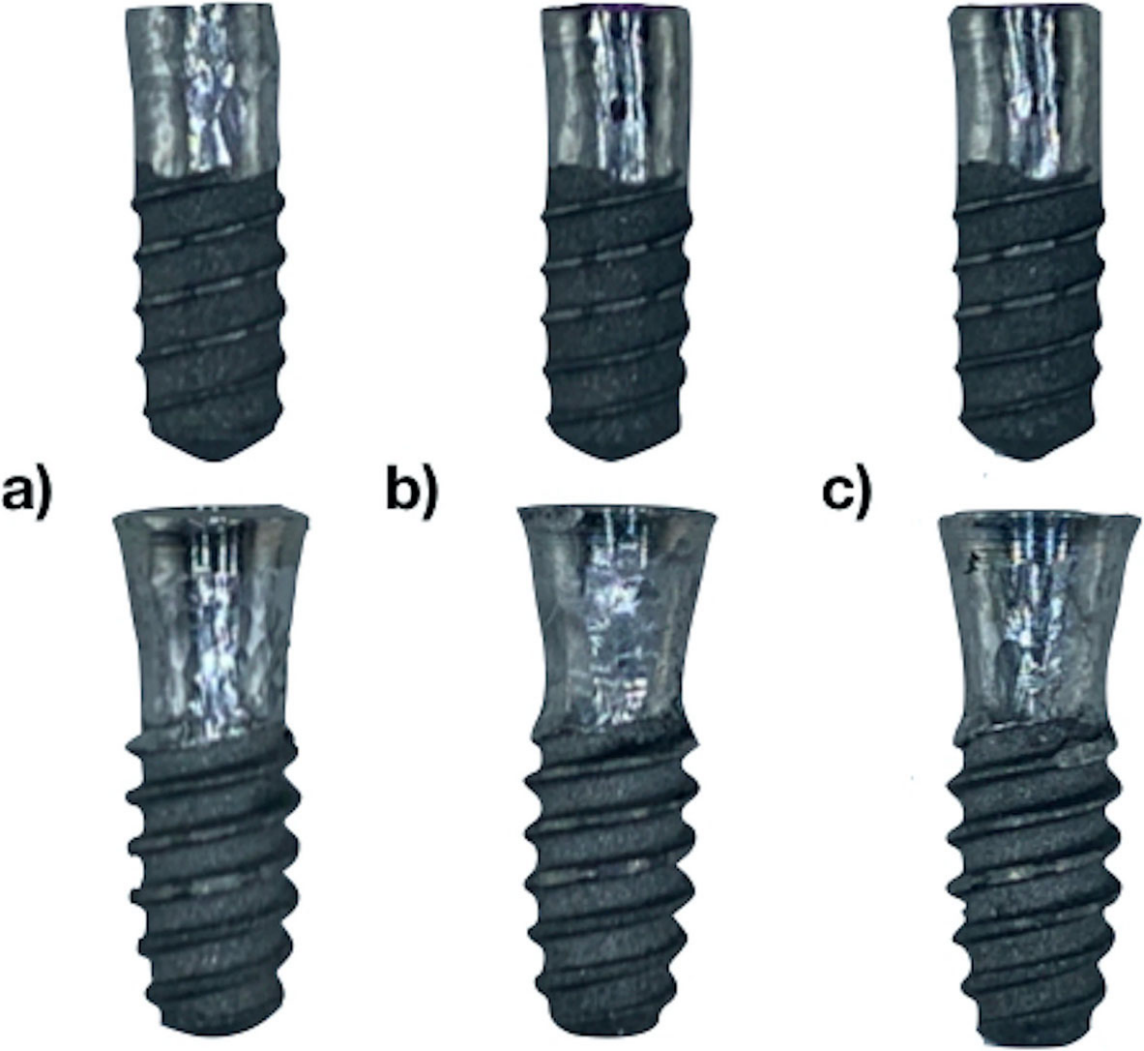
Bone-level and machined collar (tissue-level) implants with a diameter of 3.5 mm after performing a manual implantoplasty. **a** After using an oval tungsten carbide bur; **b** after surface polishing using a brown silicon carbide polisher; **c** after surface polishing using a green silicon carbide polisher

In the IMP2 group, implantoplasty was performed using Panamax 2 turbine (NSK Dental Spain SA, Madrid, Spain) with copious irrigation and a 2.5× magnifying loupe (Zeiss, Oberkochen, Germany) to simulate a typical clinical situation. In this group, pressure against the implant was not controlled. All manual implantoplasty procedures were performed by the same operator (M.D.P.). The grinding and polishing process in this group was based on the method recently described by Costa-Berenguer et al. [28]. Firstly, 3 mm of the exposed threads in the coronal area was removed using an oval tungsten carbide bur (H379 314 023; Komet Dental, Lemgo, Germany). Subsequently, a surface polishing sequence was performed using two silicon carbide polishers (9618 314 030 and 9608 314 030; Komet Dental) until a shiny clean surface was achieved (Fig. 1). A new set of burs was used for each implant. After completion of the polishing process, titanium and rubber residue were cleaned using a saline solution, and the implant block was carefully packaged.

**Fig. 2:**
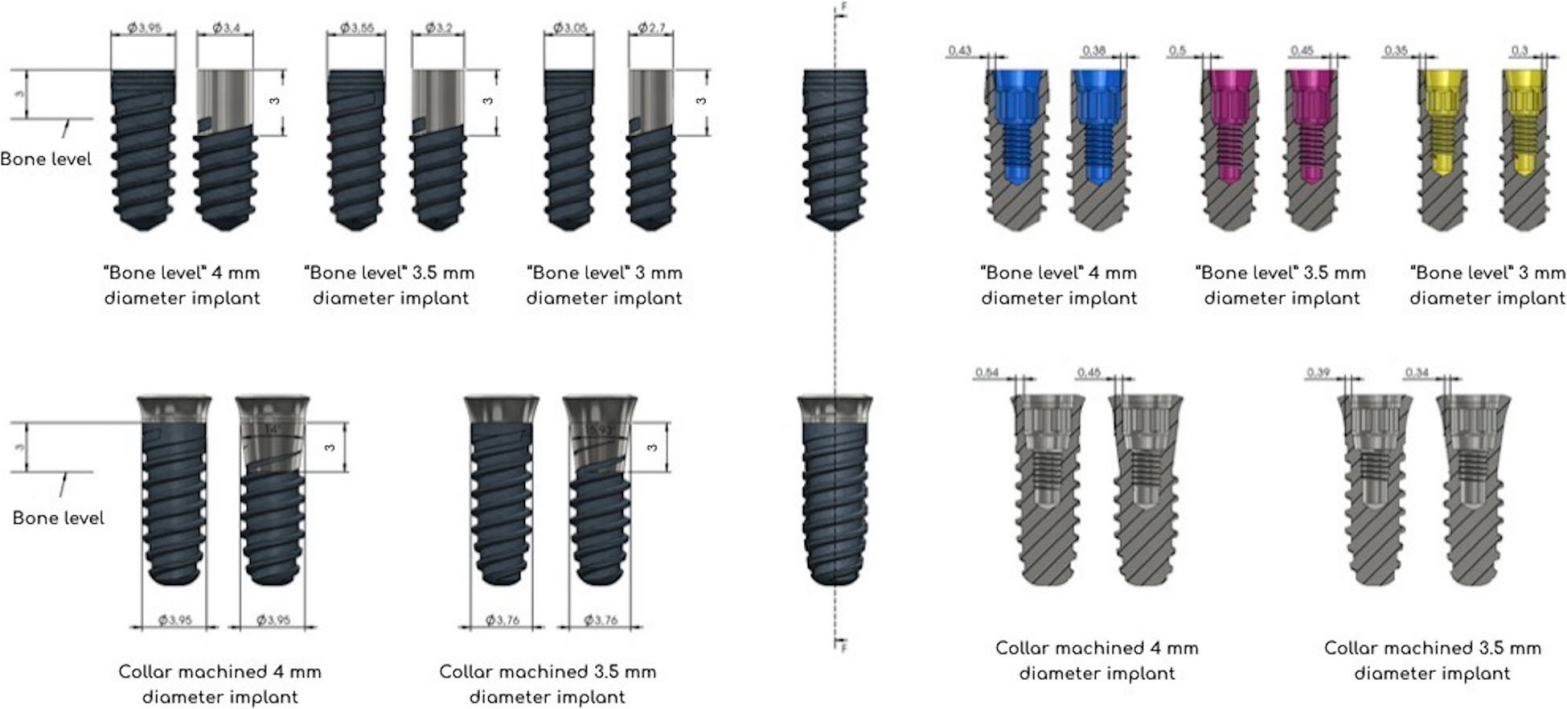
Diagram showing the steps performed for industrially machined implantoplasty (IMP1) in implants with different designs and diameters

In the IMP1 group, the implant was fixed in a resin block in the same way as the IMP2 group, and controlled milling was performed using an industrial machine (Deco 2000, Tornos Technologies Iberica, Granollers, Spain). The pressure of the drill on the implant surface was maintained at 5 Ncm, and procedure was limited to the removal of threads for each type of implant by irrigation with water and oil (Fig. 2).

### Loading tests

**Fig. 3:**
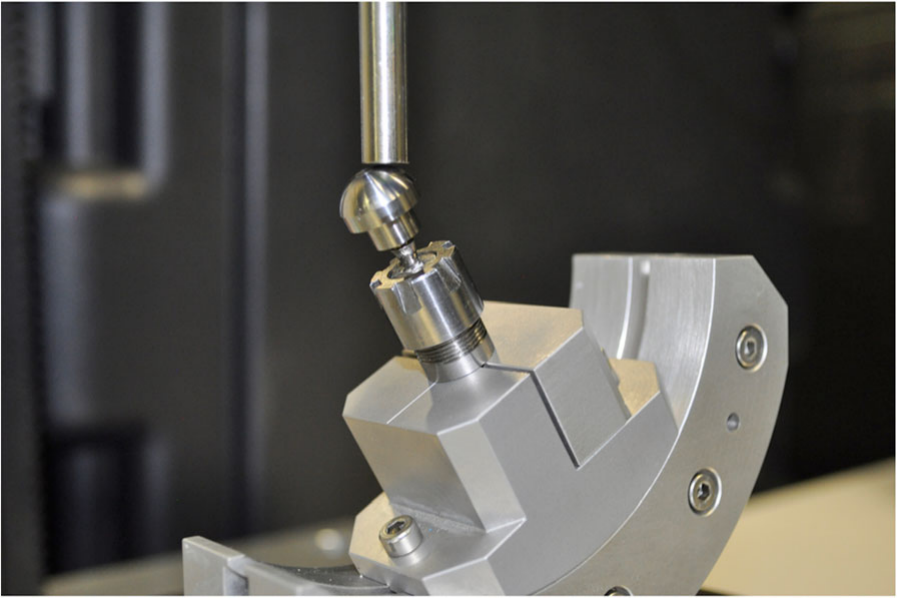
Implant with a 3.5-mm diameter held in a stainless-steel clamp with the abutment fastened and ready for the loading test

All implants were subjected to loading tests according to the ISO 14801:2016 guidelines. Universal abutments were screwed at 30 Ncm on all implants using a calibrated torque wrench (Klockner). All samples were held with the same stainless-steel clamp. Finally, compression force was applied at a constant angle of 30° from the vertical axis, and at a constant speed of 0.1 mm/s using a CNC servohydraulic machine (DYNA Mess, Stolberg, Germany; Fig. 3).

The Testworks software (MTS Systems, Eden Prairie, MN, USA) was used for recording the real-time data for all samples. After the data were obtained, the maximum compressive force tolerated by each implant was selected as its resistance to compression before exceeding its elastic limit. After the loading tests were completed, deformation and fracture shapes were analyzed.

### Evaluation of implant surfaces

After completion of implantoplasty for both groups, a randomly chosen surface of each implant was analyzed using scanning electron microscope (S-3400N; Hitachi, Tokyo, Japan) at 25×, 200×, 800×, and 2000× magnification, to identify any residue, fissures, fractures, scratches, or anomalies on the implant surface.

### Macroscopic changes in the implants

All implants were measured at the mid-zone of the implantoplasty surface (3 mm from the platform for tissue-level implants, and 1.5 mm from the platform for bone-level implants) using a micrometric precision caliper (Sylvac Cal Pro, Yverdon-les-Bains, Switzerland), to compare the diameters across the IMP1, IMP2, and control groups.

### Statistical analysis

The data were collected and analyzed using the GraphPad Prism version 7.00 software for Windows (GraphPad Software Inc., La Jolla, CA, USA). Differences in elastic limit and diameter of the implants were compared between the IMP1, IMP2, and the control groups using a Tukey’s test for multiple comparisons. A *p* value of < 0.05 was considered reflective of statistical significance.

## Results

### Loading tests

**Fig. 4:**
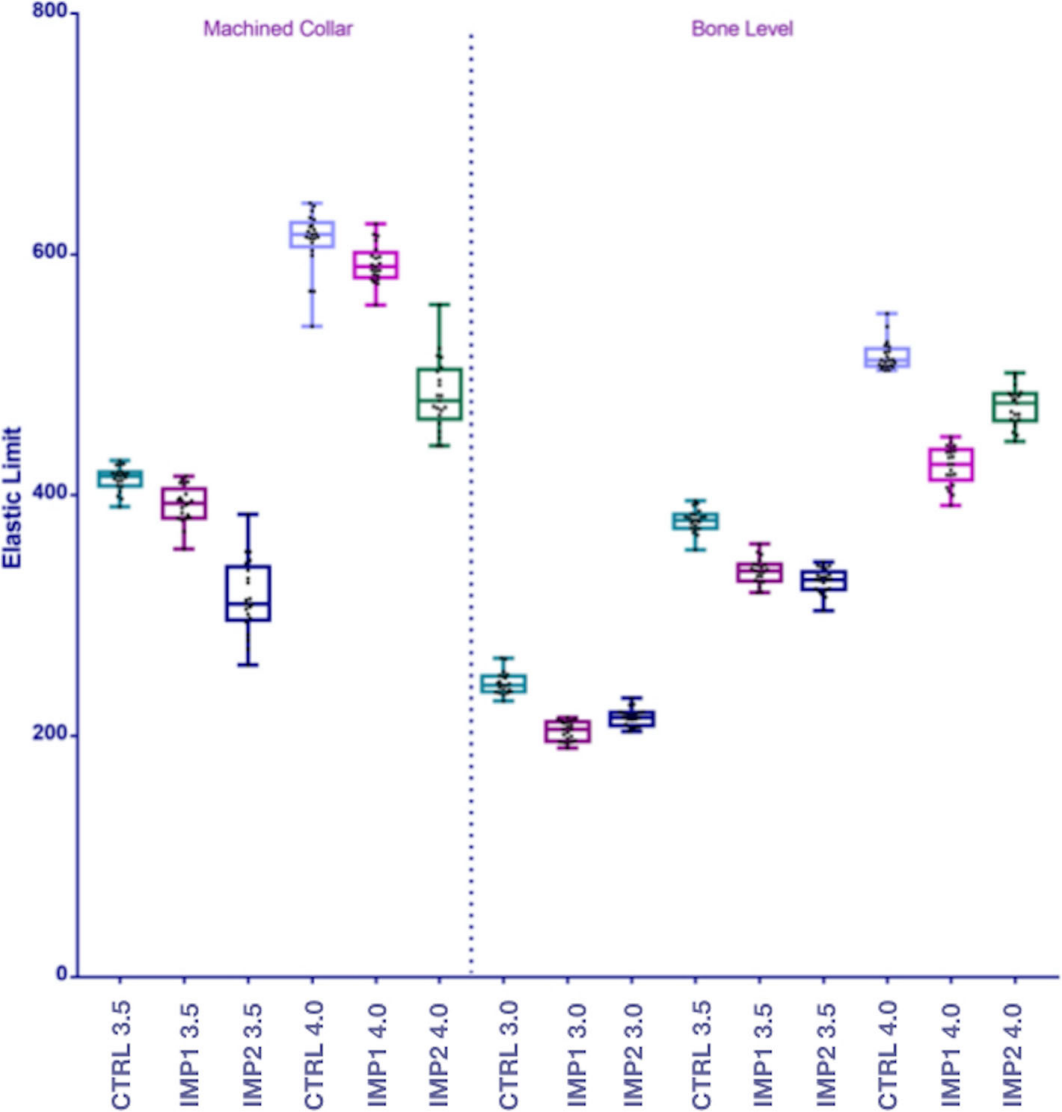
Boxplot graphs showing the elastic limit values for the machined collar (tissue-level) and the bone-level implant groups after the loading test

The data are presented as two boxplot graphs in Fig. 4. The mean elastic limit values and their standard deviations for all groups are shown in Table 1. The elastic limit reduction for tissue implants of diameters 3.5 mm and 4 mm was 5.17% and 3.18%, respectively, in the IMP1 group and 23.74% and 20.98%, respectively, in the IMP2 group . In all cases, the differences between the experimental groups and the control group were statistically significant (*p* < 0.05). Higher resistance values were observed in the IMP1 implants, with a mean difference of 76.80 N and 108.90 N for implants with diameters of 3.5 and 4 mm, respectively. Both differences were statistically significant (*p* < 0.05).

**Table 1:** Mean elastic limits for different implant diameters and designs in all study groups

		Elastic limit		
	Diameter, mm	IMP1	Control	IMP2
**Machined collar**	**3.5**	392.32 (15.26)*	413.71 (9.98)*	315.52 (30.56)*
	**4.0**	592.39 (16.01)*	611.86 (24.93)*	483.52 (29.67)*
**Bone level**	**3.0**	203.91 (7.95)*	244.03 (9.33)*	215.49 (7.20)*
	**3.5**	337.16 (10.52)*	379.04 (9.42)*	329.11 (10.11)*
	**4.0**	424.70 (15.99)*	516.15 (12.04)*	473.51 (15.92)*

The data are presented as the mean with the standard deviation in parentheses. All values are in Newtons. IMP1, industrially machined implant group; IMP2, manually polished implant group. *Statistically significant difference (*p* < 0.05)

Compared to the control group, bone-level implants in the IMP1 and IMP2 groups showed a reduction of 16.43% and 11.68%, respectively, in the elastic limit of implants with a 3 mm diameter, 11.03% and 13.17%, respectively, for implants with a 3.5 mm diameter, and 17.72% and 8.27%, respectively, for implants with a 4 mm diameter. Higher resistance values were observed in bone-level implants of diameters 3 mm and 4 mm in the IMP2 group, with respective mean differences of 11.60 N and 48.80 N. In contrast, higher resistance values were observed for implants of diameter 3.5 mm in the IMP1 group (mean difference, 8.1 N). In all cases, the differences across groups were statistically significant (*p* < 0.05).

**Fig. 5:**
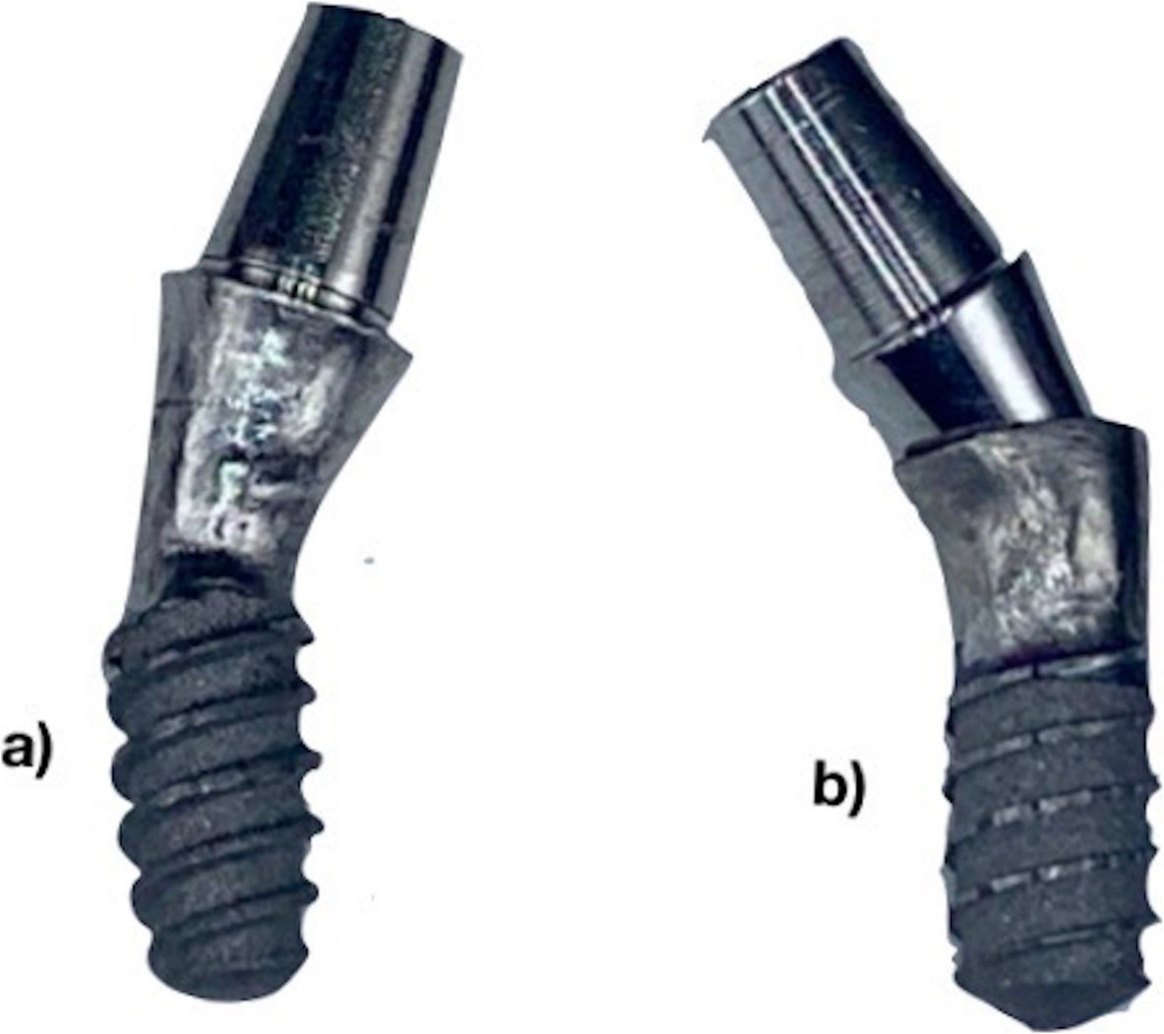
Different patterns of deformation after load testing in implants that underwent implantoplasty. **a** The tissue-level type (at the apical area of the implantoplasty). **b** The bone-level type (at the implant-abutment connection area)

All tested bone-level implants were mostly deformed in the coronal area close to the abutment connection, whereas tissue-level implants were deformed in the apical area of the surface on which implantoplasty was performed (Fig. 5).

### Evaluation of implant surfaces

**Fig. 6:**
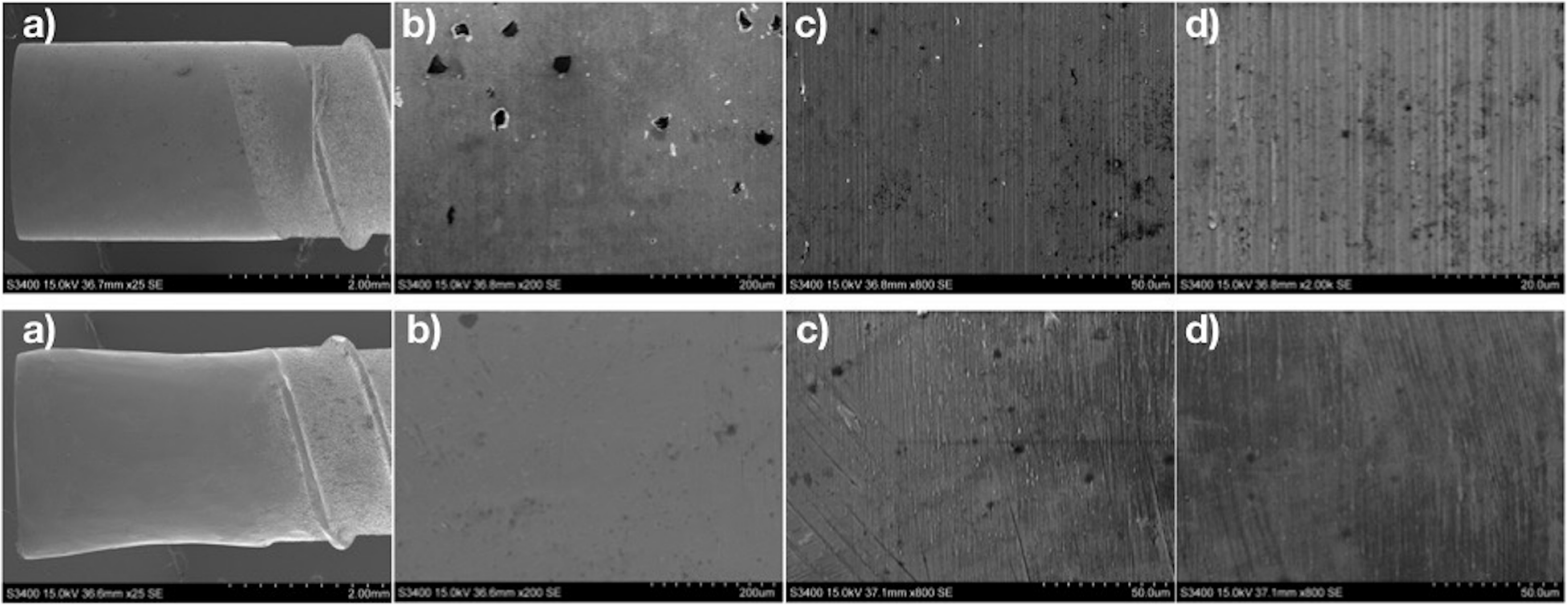
Scanning electron micrographs of a bone-level implant with industrially machined implantoplasty (IMP1) (upper images) and with manual implantoplasty (IMP2) (lower images) at (**a**) 25×, (**b**) 200×, (**c**) 800×, and (**d**) 2000× magnification. Titanium particles can be observed in the IMP1 group images at magnifications of 200× and 800×

The implant surface in the IMP2 group showed an irregular topography at a magnification of 25×; however, at higher magnification, a polished surface was seen. The implants in the IMP1 group had titanium particles over their surface (Fig. 6).

### Macroscopic analysis

**Fig. 7:**
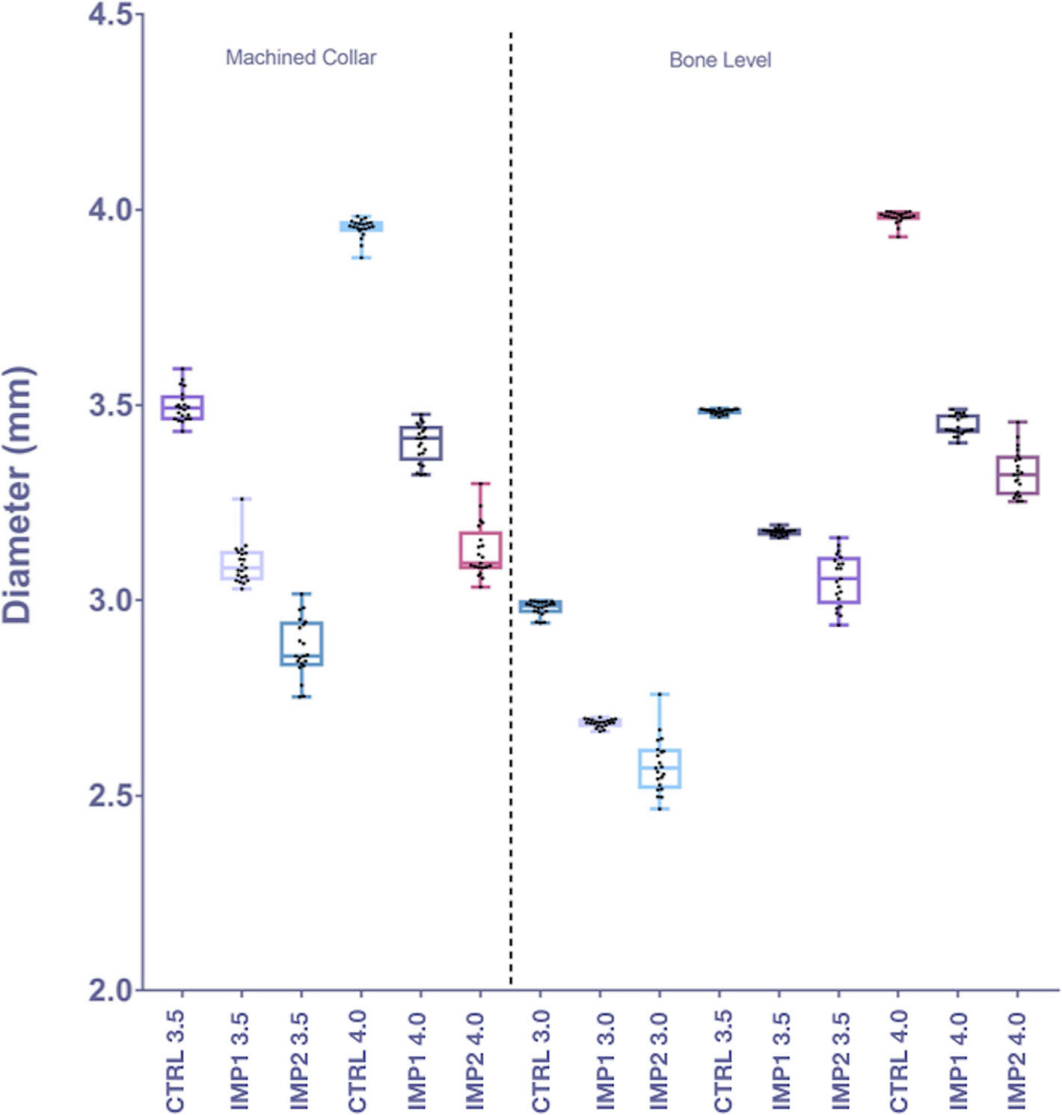
Boxplot graphs showing the data for the IMP1 and IMP2 groups with machined collar and bone-level implants after implantoplasty and the control groups according to diameter

The mean implant diameters in the IMP1, IMP2, and control groups are shown in Table 2. The data are shown as a boxplot graph in Fig. 7. The differences across groups for each implant diameter and design were statistically significant (*p* < 0.05).

**Table 2:** Mean diameters for the different types of implant design measured at the center of the surface over which implantoplasty was performed

		Diameter of the implant at the mid-zone of the surface with implantoplasty		
	Diameter, mm	IMP1	Control	IMP2
**Machined collar**	**3.5**	3.09 (0.05)*	3.55 (0.02)*	2.88 (0.07)*
	**4.0**	3.40 (0.05)*	3.97 (0.01)*	3.13 (0.07)*
**Bone level**	**3.0**	2.69 (0.01)*	2.99 (0.01)*	2.58 (0.07)*
	**3.5**	3.18 (0.01)*	3.49 (0.01)*	3.05 (0.07)*
	**4.0**	3.45 (0.03)*	3.91 (0.02)*	3.33 (0.06)*

In the control group, the measurement was taken in the corresponding area. The data are presented as the mean with the standard deviation in parentheses. IMP1, industrially machined implant group; IMP2, manually polished implant group. *Statistically significant difference (*p* < 0.05)

## Discussion

In the present study, we found a statistically significant reduction in resistance to elastic deformation in all tested implants after implantoplasty, in comparison to a control group, irrespective of whether the implantoplasty was performed manually or by industrial machining. Statistically significant differences in resistance were also found between the manually and industrially machined implants. The decrease in the elastic limit of implants with a narrower diameter could be more clinically relevant, as it could be exceeded by the occlusal force in the posterior jaw in some patients [29]. To the best of our knowledge, this is the first controlled study to compare the resistance of internal connection implants of different designs and diameters to plastic deformation (elastic limit) after being subjected to implantoplasty, including implants with a narrow diameter (3 mm). Implantoplasty poses certain problems related to the technique itself, including the lack of accessibility and professional expertise, both of which are factors that could compromise the structural integrity of the implant. This is also the first study to compare operator-related differences between the manual implantoplasty technique and a standardized industrial machining method.

No consensus remains regarding the optimal method for decontamination of implant surfaces in patients suffering from peri-implantitis [15]; however, implantoplasty has been found to be effective for plaque removal [11]. Therefore, the procedure significantly improves the clinical [18, 25] and radiological parameters in such patients [20].

Although the results of this study are specific for the implant designs used, other authors have also reported reduction in resistance to deformation in implants treated with implantoplasty. Gherke et al. [24] compared the resistance to fatigue in implants of diameter 4 mm before and after implantoplasty. In reference to whether the connection was external hexagonal, internal hexagonal, or cone Morse, the author observed a mean reduction of 37%, 40%, and 20%, respectively, in the resistance to fatigue. Even though internal hexagonal connection showed the greatest decrease in resistance to fatigue after implantoplasty, the lowest resistance values were in the group with an external hexagonal connection (487.1 ± 3.72 N). In our study, the resistance values for 4 mm tissue-level implants and 4 mm bone-level implants (483.52 ± 29.67 N and 473.51 ± 15.92 N) were similar to that observed with internal hexagonal connection (495.7 ± 85.24 N) in the study by Gherke et al. [24]. The greater decrease in the elastic limit could be due to the connection occupying a greater space within the body of implant, leading to a greater reduction in the wall thickness of treated implants, especially in the neck region.

In our study, differences in the design of the implant necks also influenced the findings in both IMP1 and IMP2 groups. The divergent implants with a machined collar were difficult to access during implantoplasty, which could have contributed to a greater decrease in the elastic limit in the IMP2 group. However, in another study, which utilized 3.75 mm and 4.7 mm implants and an external hexagonal connection, statistically significant differences were observed between the elastic limit of narrower implants treated with implantoplasty and the controls [19]. The reduction was similar to that observed in our study. Although direct comparison of the results of the two studies is not possible due to differences in the type of connection used, this reduction should be considered when using implants of a narrower diameter.

Costa-Berenguer et al. [28] compared the fracture resistance of implants with a platform diameter of 4 mm and an external connection that underwent implantoplasty to that of implants in an untreated control group. The mean resistance value was higher in the implantoplasty group than in the control group (896 N vs*.* 880 N); however, this finding was not statistically significant. Unlike in the present study, they investigated resistance to fracture rather than resistance to deformation, which could explain the difference in the findings of both studies. Moreover, the implants analyzed by them had an external connection and the fracture sites were at the neck of the implant and at the level of connection. A difference in the type of implant connection used could explain the difference in the deformation patterns seen in these studies. In the present study, the bone-level implants were deformed at the neck area of the surface on which implantoplasty was performed, whereas the implants with a machined collar were deformed at the apical area. These observations were dissimilar to those of Costa-Berenguer et al. [28], where the fractures in external connection implants with a machined collar occurred at the level of the implant body or at the abutment.

On the other hand, despite the fact that in the IMP2 group, the implant diameters were thinner than in the IMP1 group for all five types of implants, in the bone level implants of 3.0 mm and 4.0 mm, the elastic limit was higher in the IMP2 group compared to the IMP1 group. The differences in the percentage reduction in the diameter between the IMP1 and IMP2 implant groups were between 3.07 and 3.7%. Possibly, these values may not influence the total force required to exceed the elastic limit of the implants.

Several groups have analyzed different types of burs and sequences used in the implant milling protocol. Many have concluded that use of tungsten carbide burs to remove threads resulted in the lowest rise in the temperature of an implant [30]. In addition, the rubber polishing protocol led to less rough surfaces [31]. Apart from the benefits of a lower temperature and more surface smoothness, this technique has the drawback of dispersing titanium particles and residues during milling or polishing. Therefore, some authors recommend copious irrigation after the technique [28] or isolation of the area with a rubber dam to decrease aspiration of the particles [8]. In the present study, some titanium particles were observed to have been dispersed in the IMP1 group. However, when a polishing protocol was included (in the IMP2 group), a smooth surface without residual metal particles was noted.

The statistically significant differences between the results of the IMP1 and IMP2 groups indicate that the outcome of manual implantoplasty, even when performed outside the oral cavity and in the simplest of conditions, could never be equivalent to that achieved by machined implantoplasty. Moreover, the implant threads will not be removed uniformly.

Despite these problems, implantoplasty has been demonstrated to be a successful treatment for peri-implantitis, in terms of controlling inflammation around implants with considerable bone loss. A prospective randomized controlled clinical trial by Romeo et al. [18] reported an improved bleeding index, a decreased probing depth, and significantly less gingival recession in patients with implants treated by implantoplasty than in controls, during a follow-up period of 2 years. In yet another study, they concluded that peri-implant bone loss at 3 years after resective treatment along with implantoplasty was negligible; however, the marginal bone loss reached a statistically significant value of 1.54 mm when implantoplasty was not performed [20].

It should be noted that around 80% of the implants treated with implantoplasty in these studies were in the posterior region of the jaw, where a significant decrease in the elastic limit could have clinical relevance [19]. The occlusal forces over the second molars could exceed 350 N [29]; this value is higher than the elastic limit of bone-level implants of diameter 3 mm or 3.5 mm, and tissue-level implants of 3.5 mm observed in our study. Moreover, the occlusal forces could further increase during parafunctional movements, when the possibility of force exceeding the elastic limit of the implant is even greater [29].

The lower resistance of implants of a narrow diameter treated with implantoplasty should be considered while planning the placement of the implant [11]. The risk of plastic deformation would increase if the implant is unsplinted and there is considerable bone loss. It has been reported that 3 mm of marginal bone loss had resulted in a reduction of 37.2% in the mean resistance of the implant, and the value further decreased to 53.8% for 5 mm of marginal bone loss [26]. Therefore, implants that have undergone this type of treatment should be reviewed regularly [19].

As previously mentioned, achieving an optimal smoothness of the surface of a treated implant is the main objective of the treatment. In this study, surface analysis was not performed to assess roughness. However, the milling and polishing sequence used was the same as that in the study by Costa-Berenguer et al. [28], where a mean roughness index of 0.1 ± 0.02 μm was observed. It is below the established maximum value of 0.2 μm required to minimize bacterial adhesion [32]. Another limitation of this study is that we could not follow the same milling sequence for both the machined and manual groups. Therefore, the results for milling and polishing are not strictly comparable. Future studies including multiple operators, and controls for time, pressure, and angulation of drilling, during the procedure are warranted.

## Conclusions

Considering the limitations of this in vitro study, the following conclusions can be drawn: (1) implantoplasty significantly decreases the elastic limit of internal connection titanium dental implants; (2) the elastic limit of narrower (3 mm and 3.5 mm) internal connection titanium implants is significantly lower than that of a standard diameter (4 mm), and (3) more studies that include different implant designs and diameters are needed to assess the safety and long-term prognosis of implantoplasty procedures.

## Data Availability

The datasets used and/or analyzed during the current study are available from the corresponding author on reasonable request.

## Abbreviations

IMP1: Industrially machined implant
IMP2: Manually polished implant

## References

[1] Howe MS, Keys W, Richards D (2016). Long-term (10-year) dental implant survival: a systematic review and sensitivity meta-analysis. J Dent..

[2] Lang NP, Berglundh T, Working Group 4 of Seventh European Workshop on Periodontology (2011). Periimplant diseases: where are we now?—Consensus of the Seventh European Workshop on Periodontology. J Clin Periodontol..

[3] Berglundh T, Armitage G, Araujo MG, Avila-Ortiz G, Blanco J, Camargo PM, Chen S, Cochran D, Derks J, Figuero E, Hämmerle CHF, Heitz-Mayfield LJA, Huynh-Ba G, Iacono V, Koo KT, Lambert F, McCauley L, Quirynen M, Renvert S, Salvi GE, Schwarz F, Tarnow D, Tomasi C, Wang HL, Zitzmann N (2018). Peri-implant diseases and conditions: consensus report of workgroup 4 of the 2017 World Workshop on the Classification of Periodontal and Peri-Implant Diseases and Conditions. J Periodontol..

[4] Rakic M, Galindo-Moreno P, Monje A, Radovanovic S, Wang HL, Cochran D, Sculean A, Canullo L (2018). How frequent does peri-implantitis occur? A systematic review and meta-analysis. Clin Oral Invest..

[5] Pommer B, Haas R, Mailath-Pokorny R (2016). Periimplantitis treatment: long-term comparison of laser decontamination and implantoplasty surgery. Implant Dent.

[6] Esposito M, Grusovin MG, Worthington HV (2012). Interventions for replacing missing teeth: treatment of peri-implantitis. Cochrane Database Syst Rev..

[7] Sharon E, Shapira L, Wilensky A, Abu-Hatourn R, Smidt A (2013). Efficiency and thermal changes during implantoplasty in relation to bur type. Clin Implant Dent Relat Res..

[8] Aljateeli M, Fu JH, Wang HL (2012). Managing peri-implant bone loss: current understanding. Clin Implant Dent Relat Res..

[9] Renvert S, Roos-Jansaker AM, Claffey N (2018). Non-surgical treatment of peri-implant mucositis and peri-implantitis: a literature review. J Clin Periodontol..

[10] Persson GR, Samuelsson E, Lindahl C, Renvert S (2010). Mechanical non-surgical treatment of peri-implantitis: a single-blinded randomized longitudinal clinical study. II. Microbiological results. J Clin Periodontol.

[11] Suarez F, Monje A, Galindo-Moreno P, Wang HL (2013). Implant surface detoxification: a comprehensive review. Implant Dent..

[12] Mombelli A, Lang NP (2000). The diagnosis and treatment of peri-implantitis. Periodontol..

[13] Schou S, Berglundh T, Lang NP (2004). Surgical treatment of peri-implantitis. Int J Oral Maxillofac Implants..

[14] Singh P (2011). Understanding peri-implantitis: a strategic review. J Oral Implantol..

[15] Hakki SS, Tatar G, Dundar D, Demiralp B (2017). The effect of different cleaning methods on the surface and temperature of failed titanium implants: an in vitro study. Lasers Med Sci..

[16] Burgers R, Gerlach T, Hanhel S, Schwarz F, Handel G, Gosau M (2010). In vivo and in vitro biofilm formation on two different titanium implant surfaces. Clin Oral Implants Res..

[17] Rimondini L, Fare S, Brambilla E (1997). The effect of surface roughness on early in vivo plaque colonization on titanium. J Periodontol..

[18] Romeo E, Ghisolfi M, Murgolo N, Chiapasco M, Lops D, Vogel G (2005). Therapy of peri-implantitis with resective surgery. A 3-year clinical trial on rough screw-shaped oral implants. Part I: clinical outcome. Clin Oral Implants Res..

[19] Chan HL, Oh WS, Ong HS, Fu JH, Steigmann M, Sierraalta M, Wang HL (2013). Impact of implantoplasty on strength of the implant-abutment complex. Int J Oral Maxillofac Implants.

[20] Romeo E, Lops D, Chiapasco M, Ghisolfi M, Vogel G (2007). Therapy of peri-implantitis with resective surgery. A 3-year clinical trial on rough screw-shaped oral implants. Part II: radiographic outcome. Clin Oral Implants Res..

[21] Ramel CF, Lussi A, Ozcan M, Jung RE, Hammerle CH, Thoma DS (2016). Surface roughness of dental implants and treatment time using six different implantoplasty procedures. Clin Oral Implants Res..

[22] Rimondini L, Ciocagni Simoncini F, Carrassi A (2000). Micro-morphometric assessment of titanium plasma-sprayed coating removal using burs for the treatment of peri-implant disease. Clin Oral Implants Res..

[23] Matarasso S, Iorio Siciliano V, Aglietta M, Andreuccetti G, Savli GE (2014). Clinical and radiographic outcomes of a combined resective and regenerative approach in the treatment of peri-implantitis: a prospective case series. Clin Oral Implants Res..

[24] Gehrke SA, Aramburu Junior JS, Dedavid BA, Shibli JA (2016). Analysis of implant strength after implantoplasty in three implant-abutment connection designs: an in vitro study. Int J Oral Maxillofac Implants.

[25] Schwarz F, Sahm N, Iglhaut G, Becker J (2011). Impact of the method of surface debridement and decontamination on the clinical outcome following combined surgical therapy of peri-implantitis: a randomized controlled clinical study. J Clin Periodontol..

[26] Gherke SA, Souza Dos Santos Vianna M, Dedavid BA (2014). Influence of bone insertion level of the implant on the fracture strength of different connection designs: an in vitro study. Clin Oral Invest..

[27] Stavropoulos A, Bertl K, Eren S, Gotfredsen K (2019). Mechanical and biological complications after implantoplasty-a systematic review. Clin Oral Implants Res..

[28] Costa-Berenguer X, Garcia-Garcia M, Sanchez-Torres A, Sanz-Alonso M, Figueiredo R, Valmaseda-Castellon E (2018). Effect of implantoplasty on fracture resistance and surface roughness of standard diameter dental implants. Clin Oral Implants Res..

[29] Ogawa T, Suzuki T, Oishi N, Zhang X, Naert I, Sasaki K (2011). Tactile sensation and occlusal loading condition of mandibular premolars and molars. Odontology.

[30] De Souza Junior JM, Oliveira de Souza JG, Pereira Neto AL, Iaculli F, Piattelli A, Bianchini MA (2016). Analysis of effectiveness of different rotational instruments in implantoplasty: an in vitro study. Implant Dent..

[31] Englezos E, Cosyn J, Koole S, Jacquet W, De Bruyn H (2018). Resective treatment of peri-implantitis: clinical and radiographic outcomes after 2 years. Int J Periodontics Restorative Dent..

[32] Bollen CM, Papaioanno W, Van Eldere J, Schepers E, Quirynen M, Van Steenberghe D (1996). The influence of abutment surface roughness on plaque accumulation and peri-implant mucositis. Clin Oral Implants Res..

